# Phospho-dependent and phospho-independent interactions of the helicase UPF1 with the NMD factors SMG5–SMG7 and SMG6

**DOI:** 10.1093/nar/gku578

**Published:** 2014-07-10

**Authors:** Sutapa Chakrabarti, Fabien Bonneau, Steffen Schüssler, Elfriede Eppinger, Elena Conti

**Affiliations:** Max Planck Institute of Biochemistry, Structural Cell Biology Department, Am Klopferspitz 18, D-82152 Martinsried, Germany

## Abstract

Nonsense-mediated mRNA decay (NMD) is a eukaryotic surveillance pathway that recognizes mRNAs with premature stop codons and targets them for rapid degradation. Evidence from previous studies has converged on UPF1 as the central NMD factor. In human cells, the SMG1 kinase phosphorylates UPF1 at the N-terminal and C-terminal tails, in turn allowing the recruitment of the NMD factors SMG5, SMG6 and SMG7. To understand the molecular mechanisms, we recapitulated these steps of NMD *in vitro* using purified components. We find that a short C-terminal segment of phosphorylated UPF1 containing the last two Ser-Gln motifs is recognized by the heterodimer of SMG5 and SMG7 14–3–3-like proteins. In contrast, the SMG6 14–3–3-like domain is a monomer. The crystal structure indicates that the phosphoserine binding site of the SMG6 14–3–3-like domain is similar to that of SMG5 and can mediate a weak phospho-dependent interaction with UPF1. The dominant SMG6–UPF1 interaction is mediated by a low-complexity region bordering the 14–3–3-like domain of SMG6 and by the helicase domain and C-terminal tail of UPF1. This interaction is phosphorylation independent. Our study demonstrates that SMG5–SMG7 and SMG6 exhibit different and non-overlapping modes of UPF1 recognition, thus pointing at distinguished roles in integrating the complex NMD interaction network.

## INTRODUCTION

The degradation of eukaryotic mRNAs is an important mechanism in the regulation of gene expression. It allows the cell to rapidly change protein expression levels in response to intracellular or extracellular signals. In addition, mRNA decay pathways serve to maintain the fidelity of gene expression by targeting aberrant transcripts that would otherwise give rise to unphysiological and potentially deleterious protein products [reviewed in (1,2)]. A well-known example is nonsense-mediated mRNA decay (NMD), which detects and degrades mRNA transcripts containing a premature termination codon (PTC) [reviewed in (3–6)]. PTCs arise from errors in transcription and RNA processing, and from mutations in the genome. Additionally, NMD also regulates the levels of diverse cellular mRNAs, accounting for ∼5–10% of the transcriptome [reviewed in (7)].

The NMD pathway was originally discovered in yeast (8) and operates in all eukaryotes studied to date [reviewed in (9,10)]. The prevailing model of NMD postulates that a ribosome stalled at a PTC terminates translation in an aberrant manner due to adjacent *cis*-acting elements that are not present in the context of normal termination, leading to the assembly of *trans*-acting NMD factors that prompt the degradation of the PTC-mRNA [reviewed in (6,11–14)]. The predominant and best-characterized *cis*-acting element is the mammalian exon junction complex (EJC), a ribonucleoprotein particle (RNP) that assembles on the open-reading frame of spliced mRNAs (15). If present downstream of a PTC, the EJC acts in synergy with the stalled ribosome to recruit NMD factors.

In humans, several NMD factors have been identified to date (SMG1-SMG9, DHX34 and NAG/NBAS). Of these, SMG1–SMG7 were first isolated in *Caenorhabditis elegans* and named after the original mutant phenotypes (suppressor with morphogenic effect on genitalia) (16–21). More recently, a genome-wide RNA interference screen in *C. elegans* led to the identification of the two new NMD genes, *smgl*-1 and *smgl*-2, that encode for the proteins NAG/NBAS and DHX34 (22). Finally, the proteins SMG8 and SMG9 were characterized in humans as regulatory co-factors of the protein kinase SMG1 (23). The core of the NMD machinery comprises the three up-frameshift proteins UPF1, UPF2 and UPF3 (corresponding to SMG2, SMG3 and SMG4), which are conserved from yeast to humans (24–26). UPF1 is an RNA helicase essential for NMD (17,20,27,28). The catalytic activity of UPF1 is stimulated upon formation of the UPF1–UPF2–UPF3 (UPF) complex (29) and is thought to remodel the PTC-mRNA (30). In mammals, the UPF complex also binds directly to the EJC (29,31–33) and impacts on UPF1 phosphorylation (34).

The phosphorylation and dephosphorylation cycle of UPF1 is essential for NMD in metazoans (18,35). UPF1 is phosphorylated by the phosphoinositide 3-kinase related kinase SMG1 (18,21,36–39). SMG1 associates with two co-factors, SMG8 and SMG9 (23,40) and with the ribosome release factors eRF1 and eRF3 (34). In turn, phosphorylated UPF1 recruits SMG5, SMG6 and SMG7. These three proteins share a phosphoserine-binding domain similar to that found in 14–3–3 proteins (41). SMG5 and SMG7 form a stable heterodimer that binds phosphorylated residues in the C-terminus of UPF1 (38,42). SMG5 and SMG7 also mediate UPF1 dephosphorylation (18,35) and target the PTC-mRNA to sites of 5′–3′ RNA degradation known as P bodies (43). SMG6 is not part of the SMG5–SMG7 complex (35,43,44) and has distinct functional modules: it contains two EJC-binding motifs or EBMs (45) and harbors an endonuclease activity that cleaves the PTC-mRNA in proximity of the ribosome (46–48). SMG6 has been shown to bind the phosphorylated N-terminus of UPF1 in co-immunoprecipitation (co-IP) assays (38), but contradictory data from tethering assays have shown that the N-terminus of UPF1 is dispensable (42).

Elucidating the macromolecular interactions between phosphorylated UPF1 and the downstream SMG5–SMG7 and SMG6 factors is an important step toward understanding the mechanisms that bring about NMD. We set out to recapitulate the interactions *in vitro* using the available knowledge from co-IP studies with endogenous proteins. To this end, we reconstituted *in vitro* phosphorylated UPF1 and used it in biochemical assays. We show that SMG6 differs substantially from SMG5–SMG7 in the way it recognizes UPF1. We solved the crystal structure of the SMG6 TPR domain and elucidate the structural basis for the differences in the functions of SMG6 and SMG5–SMG7.

## MATERIALS AND METHODS

### Protein expression and purification

His- and His-GST tagged SMG6 TPR were expressed using *Escherichia coli* BL21-Gold (DE3) pLysS cells (Stratagene) grown in TB medium and induced overnight at 18°C. The cells were lysed in buffer A (20 mM Tris-HCl pH 7.5, 10 mM imidazole, 10% glycerol) supplemented with 200 mM NaCl and protease inhibitors (Roche). The proteins were purified by Ni^2+^- affinity chromatography as an initial step and further purified over a HiTrap Q Sepharose HP column (GE Healthcare) to remove minor contaminants. The His-tag was removed by treatment with Tobacco Etch Virus (TEV) protease for crystallization studies. Size-exclusion chromatography (SEC) on a Superdex 200 column (GE Healthcare) was performed as a final step of purification using buffer B (20 mM HEPES pH 7.5 and 2 mM DTT) supplemented with 100 mM NaCl and 2% glycerol. Selenomethionine substituted protein was purified as described above from *E. coli* grown in M9 media complemented with the essential amino acids and selenomethionine (49).

In order to purify the SMG5–7 TPR complex, His-(TEV)-SMG7 and SMG5-His were co-expressed in *E. coli* BL21 (DE3) STAR pRARE cells, which were grown and induced as described above. The cells were lysed in buffer A containing 250 mM NaCl. The proteins were purified by Ni^2+^- affinity chromatography and subjected to treatment with TEV protease overnight. A second Ni^2+^- affinity column was performed to remove the excess SMG7 TPR, which was further purified by SEC. Anion-exchange chromatography (HiTrap Q HP Sepharose) yielded a stoichiometric SMG5–7 TPR complex. The complex was stored in buffer B supplemented with 300 mM NaCl and 10% glycerol.

GST-UPF1 constructs were expressed in *E. coli* BL21 (DE3) STAR pRARE cells as described above. The cells were lysed in buffer A supplemented with 500 mM NaCl, 0.1% NP-40 detergent and protease inhibitors and additionally with 1 μM ZnCl_2_ and 1 mM MgCl_2_ for constructs encompassing the CH and helicase domains. The proteins were purified by sequential Ni^2+^- affinity and ion-exchange chromatography (HiTrap Q/Heparin HP, depending on the pI of the construct) and a final step of SEC, using a S200 column in buffer B containing 150 mM NaCl and 10% glycerol. All UPF1 mutants were engineered using the Stratagene Quikchange kit and verified by DNA sequencing.

The proteins SMG6fl, SMG1 wt and SMG1 KD were designed to contain an N-terminal HA-Flag tag and were transiently expressed and purified from mammalian cells by Flag-affinity chromatography. HEK 293T cells were cultured in Dulbecco's modified Eagle medium containing 10% fetal bovine serum (Gibco), 2 mM L-glutamine, 1000 U/ml penicillin and 0.1 mg/ml streptomycin (Gibco) and transfected with mammalian expression plasmids using polyethyleneimine ‘Max’ (Polysciences Inc., Mw 40000). The transfected cells were incubated at 32°C for 72 h. The cells were then resuspended in lysis buffer (1.5× phosphate buffered saline, 1 mM MgCl_2_, 1 mM CHAPS and 10% glycerol) supplemented with 0.5 μg/ml DNase I, 60 μg/ml RNase A and protease inhibitors and incubated for 15 min. The lysate was centrifuged at 17,000 × g for 15 min at 4°C. The clarified cell lysate was incubated with anti-Flag M2 resin (Sigma) at 4°C for an hour. After extensive washing to remove non-specifically bound proteins, the Flag-tagged protein of interested was eluted from the resin with 100 μM of 1× Flag peptide (Sigma) in the same buffer.

### Crystallization and structure determination

Native crystals of SMG6 TPR were grown at 20°C by sitting-drop vapor diffusion from drops formed by equal volumes of protein (at 2 mg/ml) and of crystallization solution containing 50 mM MES pH 5.5, 200 mM CH_3_COONH_4_ and 2.5% 2-Methyl-2,4-pentanediol (MPD). Crystals were cryoprotected with a final concentration of 40% MPD prior to data collection. Seleno-methionine derivatised crystals were grown in similar conditions at 4°C and cryo-protected as described above.

Native and single-wavelength anomalous dispersion (SAD) data were collected at the PXII and PXIII beamlines of the Swiss Light Source (SLS) (Villigen, Switzerland), respectively. Data were processed with XDS (50) and scaled using Aimless (51). Selenium sites were first located with SHELXD (52), following which experimental phases were calculated using the AutoSol pipeline in Phenix (53). Iterative cycles of model building and refinement were carried out with COOT (54) and Phenix and the final model was validated using Molprobity (55).

### *In vitro* kinase assays

Full-length GST-UPF1 (1 μg) was incubated with catalytic amounts of either SMG1 wt or SMG1 KD proteins in kinase reaction buffer (25 mM MOPS pH 7.2, 5 mM MgCl_2_, 10 mM β-glycerophosphate, 50 μg/ml BSA) at 30°C. The phosphorylation reactions were initiated by the addition of a radiolabeled ATP mix, consisting of 1 mM ATP and 0.2 μCi of γ-^32^P ATP and allowed to proceed for 1 h. The reactions were quenched by the addition of SDS sample buffer and analyzed by sodium dodecyl sulphate-polyacrylamide gel electrophoresis (SDS-PAGE) and autoradiography.

### GST pull-down assays

Experiments were performed by mixing ∼2 μg of GST-tagged (bait) protein with equal amounts of untagged (prey) protein. GST-reaction buffer (20 mM HEPES pH 7.5, 125 mM NaCl, 2% glycerol, 2 mM DTT) was added to a final volume of 45 μl. Wherever indicated, GST-UPF1 constructs were phosphorylated (as described above) prior to the addition of the prey protein. The reaction mixtures were incubated at room temperature for 45 min, following which 15 μl of 50% (v/v) suspension of Glutathione–Sepharose beads (GE Healthcare) was added to each reaction mixture. The resultant mixture was supplemented with 200 μl of binding buffer (20 mM HEPES pH 7.5, 150 mM NaCl, 10% glycerol, 2 mM DTT, 0.1% NP-40) and incubated at 4°C for 1 h. The beads were washed four times with 500 μl binding buffer. Bound proteins were eluted with 20 mM reduced glutathione, resolved by SDS-PAGE and visualized by staining with Coomassie.

### co-IP assays

For co-IP studies, HEK 293T cells (cultured as described above) were co-transfected with plasmids of HA-SMG6 constructs and Flag-His-UPF1fl using polyethyleneimine. The SMG6 constructs were also co-transfected with Flag-ILF2 as a negative control. Transfected cells were incubated at 32°C and harvested 72 h later. Cell extracts were prepared in 250 μl NET-G buffer (50 mM Tris-HCl pH 8.0, 150 mM NaCl, 0.1% NP-40, 1 mM EDTA, 10% glycerol); 12 μl of anti-Flag M2 resin (Sigma) were added to 200 μl of each co-transfected cell extract and incubated at 4°C for 1 h. The beads were washed four times with 1 ml NET-G buffer to remove non-specifically bound proteins, while Flag-tagged proteins and their interaction partners were eluted with 40 μl of non-reducing SDS sample buffer. The eluted proteins were resolved by SDS-PAGE and then transferred onto polyvinylidene difluoride membranes for analysis by western blotting. The membranes were probed with mouse monoclonal anti-HA (Covance) and anti-Flag (Sigma) antibodies to detect SMG6 and UPF1/ILF2, respectively. A horseradish peroxidase-conjugated goat anti-mouse antibody (Bio-Rad) used in combination with ECL prime western blotting detection reagent (GE Healthcare) enabled detection of the tagged proteins by chemiluminescence.

### Analytical ultracentrifugation

Sedimentation velocity experiments were performed on an Optima XL-I analytical ultracentrifuge (Beckman Inc.) using an An 60 Ti rotor and double-sector epon centerpieces. The proteins were used in analytical ultracentrifugation (AUC) buffer (20 mM HEPES pH 7.5, 150 mM NaCl, 5% glycerol) at 0.4 mg/ml. Buffer density and viscosity were measured using a DMA 5000 densitometer and an AMVn viscosimeter, respectively (both Anton Paar). Protein concentration distribution was monitored at 280 nm, at 50 000 rpm and 20°C. Time-derivative analysis was computed using the SEDFIT software package, version 12.1b (56), resulting in a c(s) distribution and an estimate for the molecular weight Mf (from the sedimentation coefficient and the diffusion coefficient, as inferred from the broadening of the sedimentation boundary, assuming all observed species share the same frictional coefficient f/f0).

## RESULTS AND DISCUSSION

### Human SMG5–SMG7 and SMG6 TPRs bind *in vitro* phosphorylated UPF1 with different efficiencies

**Figure 1.**
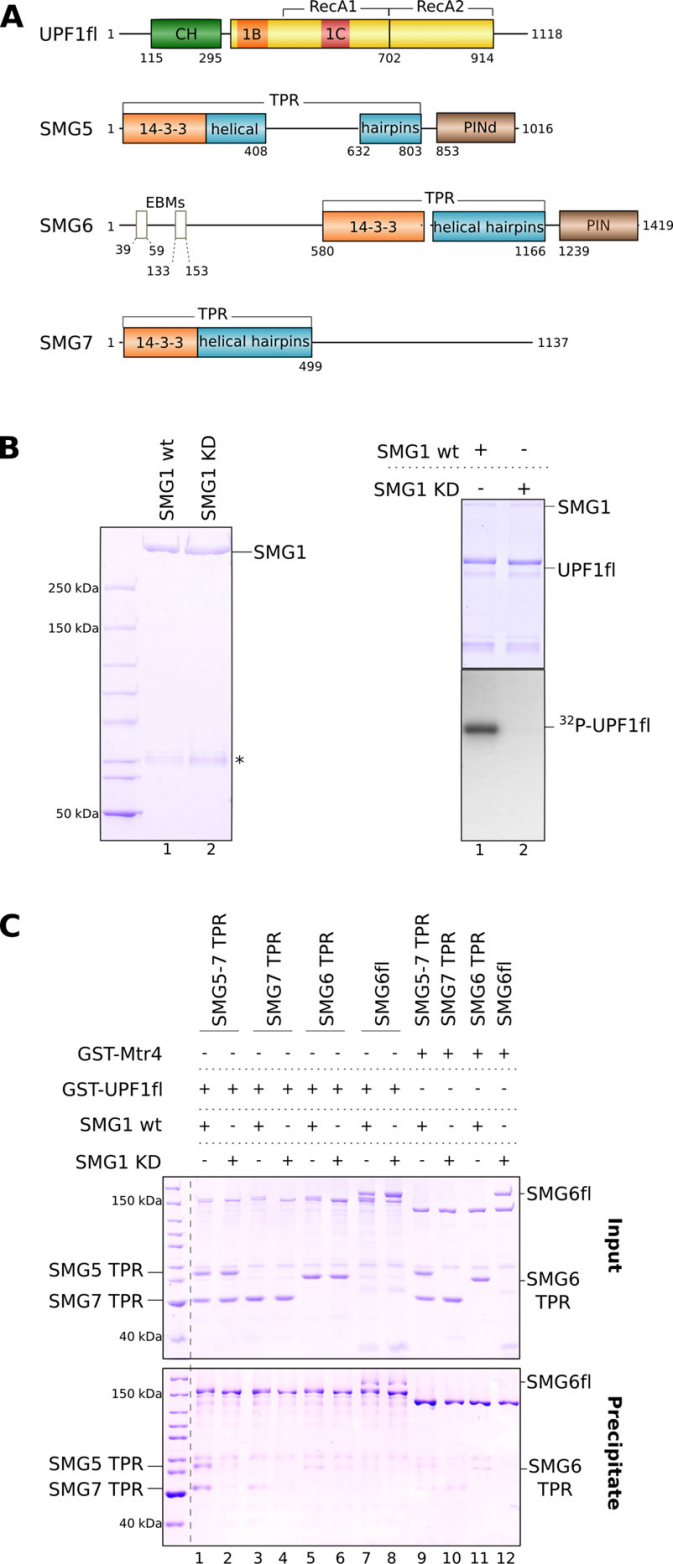
Interaction of human SMG5, SMG6 and SMG7 with *in vitro* phosphorylated UPF1. (**A**) Schematic representation of the domain organization of full-length (fl) human UPF1, SMG5, SMG6 and SMG7. Folded domains are shown as rectangles and low-complexity sequences as lines. In UPF1, the CH domain (in green) and the ATPase core domains (RecA1 and 2, 1B and 1C, colored yellow, orange and red, respectively) are indicated. The regions N- and C-terminal of the core contain the SMG1 phosphorylation sites. The TPR domains of SMG5, SMG6 and SMG7 comprise the 14–3–3 and helical hairpins domain, which are colored orange and teal, respectively. The SMG5 TPR domain is bifurcated by a long linker, which was deleted in the construct used in this study. The N-terminal EBMs of SMG6 are shown as boxes. In brown are the active and catalytically dead (d) PIN domains at the C-terminus of SMG6 and SMG5, respectively. (**B**) Left panel: SDS-PAGE analysis of catalytically active (wt) and inactive (kinase-dead, KD) SMG1 proteins purified from HEK 293T cells. The asterisk (*) indicates a contaminant (methylosome) that co-purifies with SMG1. Right panel: *in vitro* kinase assay performed using purified SMG1 proteins and UPF1fl as a substrate. A corresponding Coomassie-stained gel of the radioactive kinase assay (using γ-^32^P ATP) indicates the enzyme: substrate ratio employed in the assay. (**C**) GST pull-down assays of GST-UPF1fl (treated with active SMG1 wt or inactive SMG1 KD mutant) and SMG5–7 TPR, SMG7 TPR, SMG6 TPR and SMG6fl. GST-Mtr4 was used as a negative control in this and all other GST pull-down experiments. One-fifth of the reaction mixture was used as input. Inputs and the bound fractions (precipitates) were analyzed on 8% SDS-PAGE gels and are shown in the top and bottom panels, respectively. The SMG5–7 TPR complex showed a significant affinity toward phospho-UPF1fl while SMG6fl binds UPF1fl in a phosphorylation-independent manner. The TPR domains of SMG6 and SMG7 exhibit only a weak affinity toward UPF1fl.

UPF1 has a central helicase region with the catalytic ATPase core (consisting of the RecA1, RecA2, 1B and 1C domains) and the regulatory CH domain, which modulates binding to UPF2 as well as to RNA (Figure 1A) (29,57,58). In metazoans, the helicase region of UPF1 is flanked by N- and C-terminal tails, which can be phosphorylated at Ser-Gln (SQ) motifs by the SMG1 kinase (21,35,38). The tails regulate the helicase activity of UPF1 (59) as well as its interaction with SMG5, SMG6 and SMG7 (38,41,42). Since phosphomimetic mutants of 14–3–3 targets often do not completely reproduce the effects of phosphorylation (60), we set out to obtain the 410 kDa human SMG1 kinase in a recombinant form. We used transiently transfected HEK 293T cells to express and purify SMG1 wild-type (SMG1 wt, Figure 1B, left panel, lane 1) and, for control experiments, a SMG1 mutant containing the D2331A substitution that renders the kinase catalytically inactive (21) (SMG1 KD, Figure 1B, left panel, lane 2). Using a radioactive kinase assay, we demonstrate that SMG1 wt was able to phosphorylate UPF1fl (Figure 1B, right panel, lane 1). The phosphorylation can be specifically attributed to SMG1 since no background phosphorylation was observed with the SMG1 KD mutant (Figure 1B, right panel, lane 2). Phosphopeptide analysis combined with LC-MS/MS confirmed that UPF1 was phosphorylated by SMG1 at SQ and TQ motifs that are present exclusively in the N- and C-terminal tails (Supplementary Figure S1A). The phosphorylation sites detected by our mass-spectrometric analysis include residues Thr28, Ser1078, Ser1096 and Ser1116, which were previously identified as sites of SMG1 phosphorylation *in vivo* (21,38). Interestingly, the N- and C-terminal tails of UPF1 in isolation (without the CH and helicase core domains) are also effective substrates for SMG1 (Supplementary Figure S1B).

In order to analyze the interactions of UPF1 with the NMD factors SMG5, SMG6 and SMG7, we recombinantly expressed and purified fragments of these proteins. The domain architecture of human SMG5, SMG6 and SMG7 can be extrapolated from previous structural studies and from sequence analyses (Figure 1A). In the case of SMG7 and SMG5, the 14–3–3-like domain and the adjacent helical hairpins domain form a single unit (41,42) that will be referred to as the tetratricopeptide (TPR) region. The boundaries of the TPR region of human SMG7 (residues 1–499) are known from previous structural studies (41). Sequence analysis suggests that the TPR region of human SMG5 contains an evolutionary divergent 220 amino-acid insertion that is not present in the sequence and structure of the *C. elegans* orthologue (42). We removed the predicted insertion in human SMG5 to obtain a proteolytically-stable protein (SMG5 1–803, Δ408–632, thereafter referred to as SMG5 TPR). SMG5 TPR was mostly insoluble on its own (data not shown) but was soluble when co-expressed with SMG7 TPR. The resulting complex of the TPR domains of SMG5 and SMG7 is referred to as SMG5–7 TPR in the text. SMG6 is expected to contain a similar 14–3–3 domain that is positioned in the middle of the polypeptide chain (Figure 1A). Based on sequence analysis, we engineered a soluble construct of SMG6 TPR that spans residues 580–1166.

We performed pull-down assays with phosphorylated and unphosphorylated GST-tagged UPF1, which were generated by incubating UPF1 either with SMG1 wt or SMG1 KD (Figure 1C). Consistent with previous co-IP studies, UPF1fl co-precipitated the SMG5–7 TPR complex only when phosphorylated (Figure 1C, compare lanes 1 and 2) (38,41,42). The SMG7 TPR in isolation showed only weak binding to phospho-UPF1 in the pull-down assay (Figure 1C, lane 3, compare with lane 1). Likewise, we did not observe significant binding of SMG6 TPR to phospho-UPF1 above background (Figure 1C, lane 5). The *in vitro* pull-down assays with recombinant proteins thus show that the SMG5 and SMG7 TPR domains heterodimerize for efficient binding to phosphorylated UPF1, recapitulating the results from cell-based studies (35,38,42).

### SMG5–SMG7 binds *in vitro* a phosphorylated stretch of UPF1 containing the two C-terminal SQ motifs

**Figure 2.**
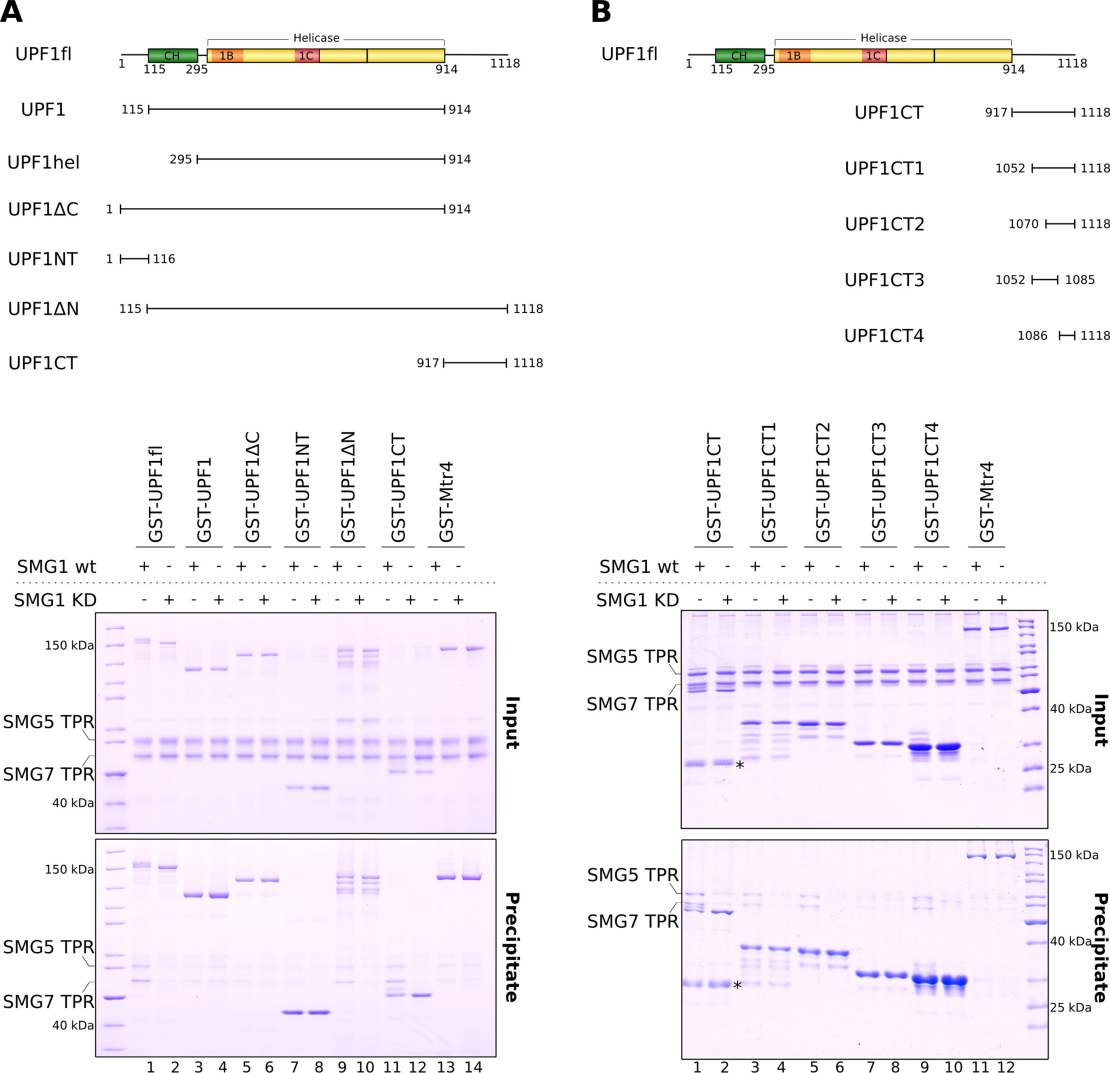
The SMG5–7 TPR complex binds the extreme C-terminal segment of UPF1. (**A**) Top panel: schematic representation of the UPF1 constructs designed to map the binding site of SMG5–7 TPR on UPF1. Bottom panel: GST pull-down assays of SMG5–7 TPR with the GST-UPF1 constructs described in the top panel. The assays were carried out in the presence of either SMG1 wt or SMG1 KD mutant as described for Figure 1C. The inputs and precipitates were analyzed on 10% SDS-PAGE gels. In the presence of SMG1 wt, the 200-residue stretch following the UPF1 helicase domain is sufficient for binding SMG5–7 TPR (see also Supplementary Figure S1B). (**B**) Top panel: schematic representation of the GST-UPF1 C-terminal constructs designed to map the SQ motifs that mediate binding of SMG5–7 TPR to UPF1. Bottom panel: GST pull-down assays (performed as described above) suggest that residues S1096 and S1116, when phosphorylated, act as interaction motifs for SMG5–7 TPR (see also Supplementary Figure S1C). The inputs and precipitates were analyzed on 15% SDS-PAGE gels. The asterisk (*) indicates a degradation product of GST-UPF1CT.

The SMG5–7 TPR heterodimer contains two potential phosphoserine binding sites, one within each 14–3–3-like domain (Figure 1A) (35,38,41). Immunoprecipitation studies have converged on Ser1096 in the C-terminal tail of UPF1 as the most important phospho site for binding to SMG7. However, the region of UPF1 required to bind SMG5 is still unknown. To address this, we designed a series of UPF1 deletion constructs, systematically lacking either or both the N-terminal and the C-terminal tail, the CH domain or the helicase core (Figure 2A, top panel). The constructs were expressed and purified as GST-fusion proteins from *E. coli*, phosphorylated *in vitro* by SMG1 and subsequently used in GST pull-down assays (Figure 2A, bottom panels). The helicase core lacking the N- and C-terminal tails did not efficiently precipitate SMG5–7 TPR (Figure 2A, lane 3). Removal of the C-terminal tail (UPF1ΔC) substantially decreased binding (Figure 2A, lane 5), while removal of the N-terminal tail had a less drastic effect (Figure 2A, lane 9). The N-terminal tail in isolation (UPF1NT) was unable to pull down SMG5–7 TPR (Figure 2A, lane 7). Instead, the C-terminal tail (UPF1CT, residues 917–1118) precipitated SMG5–7 TPR with a similar efficiency as compared to UPF1fl (Figure 2A, lane 11, compare with lane 1). As expected, the interaction was dependent on phosphorylation (Figure 2A, compare lanes 11 and 12). We concluded that the C-terminal tail of UPF1 contains the major determinants for binding the SMG5–7 TPR heterodimer.

To narrow down which portion of the UPF1 C-terminal tail is required for SMG5–7 TPR binding, we engineered a series of GST-fusion UPF1CT deletion constructs lacking specific SQ motifs (Figure 2B, top panel) and performed GST pull-down assays as before (Figure 2B, bottom panels). The constructs UPF1CT1 (residues 1052–1118) and UPF1CT2 (residues 1070–1118) recapitulated the binding of UPF1CT to SMG5–7 TPR (Figure 2B, lanes 3 and 5, compare with lane 1). UPF1CT2 is a 50-residue segment comprising five serines in SQ motifs: S1073, S1078, S1089, S1096 and S1116 (Supplementary Figure S1A). Removal of the last two SQ motifs in the UPF1CT3 construct (residues 1052–1085) impaired SMG5–7 TPR binding (Figure 2B, lane 7). The UPF1CT4 construct (residues 1086–1118) including only the last two SQ motifs was, instead, sufficient to bind SMG5–7 TPR (Figure 2B, lane 9, see also Supplementary Figure S1C). Interestingly, immunoprecipitation studies had implicated S1078 and S1116 for SMG5–7 binding, albeit with a weaker contribution than S1096 (38). The *in vitro* pull-down assays with recombinant proteins thus indicate that the segment of UPF1 centered at the strong S1096 site and at the weaker S1116 site are sufficient for binding the SMG5–7 TPR.

### SMG6 TPR is a monomeric 14–3–3-like domain

**Figure 3.**
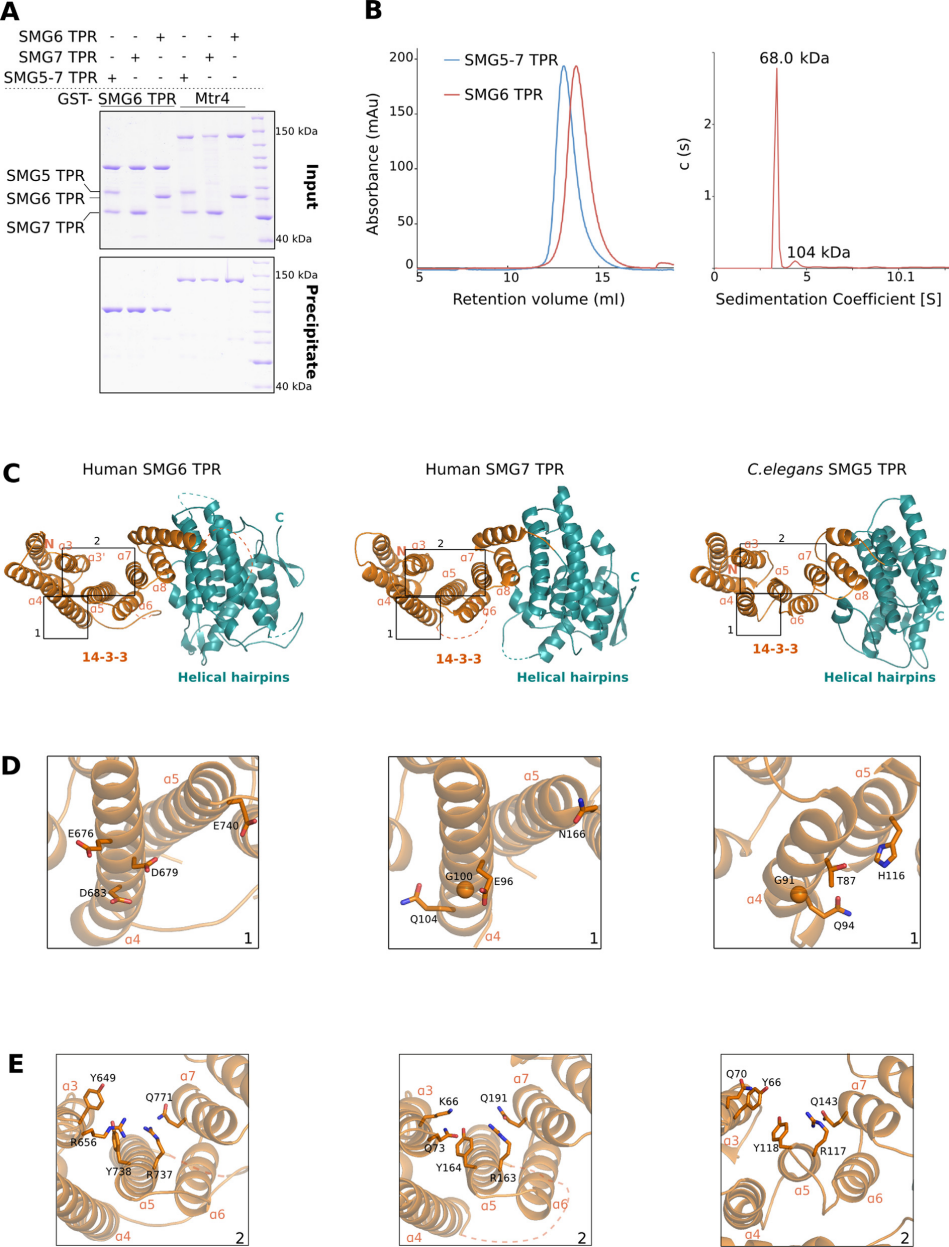
Overall structure of the SMG6 TPR domain. (**A**) GST pull-down assays of GST-SMG6 TPR with that of other TPR domains, carried out as in Figure 1C. The samples were analyzed on 10% SDS-PAGE gels; panels for inputs and precipitates are indicated. While the TPR domains of SMG5 and SMG7 interact to form a stable complex, that of SMG6 is unable to mediate such interactions. (**B**) Left panel: size-exclusion analyses of SMG5–7 TPR and SMG6 TPR proteins. The purified SMG5–7 TPR complex and SMG6 TPR were separately injected on a semi-analytical gel-filtration column (Superdex 200 HR 10/30, GE Healthcare) and the chromatograms were overlaid. The size-exclusion analyses demonstrate that SMG6 TPR is a monomer in solution (molecular weight of 68 kDa) in contrast to the dimeric SMG5–7 TPR (molecular weight of 123 kDa). Right panel: sedimentation velocity AUC of SMG6 TPR. SMG6 TPR was monitored at 280 nm and 50 000 rpm at 20°C for 16 h. The molecular weight of SMG6 was estimated from the sedimentation coefficient and the diffusion coefficient. (**C**) Overall crystal structure of the TPR domains of SMG6 (left), human SMG7 (middle, PDB ID 1YAO) and *C. elegans* SMG5 (right, PDB ID 3ZHE). The structures are shown in the same orientation after optimal superposition of their N-terminal 14–3–3 domain. The helices within the N-terminal 14–3–3 domain (colored orange) encompass the phosphoserine-binding motif while the helices of the C-terminal domain (colored teal) are arranged into helical hairpins. The highlighted region 1 depicts the hotspot of dimerization in the 14–3–3 domains, as derived from the structure of *C. elegans* SMG5–7 TPR. The highlighted region 2 indicates the phosphoserine-binding pocket within the 14–3–3 domains of the three proteins. This and all other structure figures were generated using PyMOL (http://www.pymol.org). (**D**) A close-up view of the highlighted region 1 from Figure 3C. A conserved glycine residue in helix α4 of SMG7 and SMG5 (middle and right panels) is at the center of the SMG5–7 TPR interface. The equivalent residue in helix α4 of SMG6 (left panel) is an asparate (D683, see also Supplementary Figure S3). (**E**) A close-up view of the highlighted region 2 from Figure 3C. The residues lining the phosphoserine-binding pockets of SMG6, SMG7 and SMG5 are highlighted.

Proteins of the 14–3–3 family are known to function as homo- or heterodimers (61). SMG5 and SMG7 form a heterodimer via the interaction of their 14–3–3 domains, but neither associates with SMG6 *in vivo* (35,38,41–44). Consistently, *in vitro* pull-down assays showed no interaction of GST-SMG6 TPR with either the SMG5–7 TPR complex or SMG7 TPR in isolation (Figure 3A, lanes 1 and 2). We reasoned that the SMG6 TPR might self-assemble in a homodimer, precluding its interaction with other TPR proteins. However, the GST-SMG6 TPR bait failed to interact with the untagged SMG6 TPR (Figure 3A, lane 3). To exclude that the GST tag might interfere with binding, we determined the oligomeric state of untagged SMG6 TPR by SEC and analytical ultra-centrifugation (Figure 3B, left and right panels, respectively). We found that SMG6 TPR was a monomer in solution and had no propensity for self-interaction.

To understand the basis for the monomeric nature of SMG6 TPR, we determined its structure. We obtained crystals of human SMG6 TPR that diffracted to 2.1 Å resolution and used SAD to solve the structure. The final model was refined to 2.1 Å resolution, with an *R*_work_ of 20.6%, *R*_free_ of 23.7% and good stereochemistry (data collection and refinement statistics in Table 1). The model includes residues 580–1159, with the exception of disordered loops at residues 711–715, 823–878 and 955–963. The overall structure of SMG6 TPR is similar to that of SMG7 TPR and SMG5 TPR (Figure 3C). SMG6 TPR features the typical 14–3–3 like domain (α-helices α1–α9, colored orange). It also has a C-terminal helical hairpins domain with seven α-helices (α10–α16, colored teal) that are stacked perpendicular to the 14–3–3-like domain. The helical hairpins domain of human SMG6 superposes with a root-mean-square deviation (r.m.s.d.) of 2.1 Å over 70% of the Cα atoms with respect to the corresponding domain in human SMG7. Besides differences in the relative orientation of the α-helices, the loops connecting them are generally longer in human SMG6 as compared to the helical hairpins domains of SMG7 and SMG5 (Supplementary Figure S2). Additionally, the linker between the 14–3–3-like and the helical hairpins domain is longer in SMG6 than in SMG7 (68 residues in SMG6 as compared to 11 residues in SMG7). Fifty-five of the 68 residues of this linker in SMG6 are disordered, as indicated by dotted lines in the structure (Figure 3C and Supplementary Figure S2). The 14–3–3 like domains of SMG6 and human SMG7 are instead more similar and can be superposed with an r.m.s.d. of 1.2 Å over 80% of their Cα atoms. Although the r.m.s.d. of superposition between SMG6 and SMG5 is considerably higher (2.7 Å over 65% of the Cα), the helical architecture of the 14–3–3-like domain is comparable to that of SMG5 (Figure 3C).

**Table 1.** Data collection and refinement statistics

**Data collection**		
Data set	SAD	Native
Beamline	SLS PXIII	SLS PXII
Space group	*P*2_1_2_1_2_1_	*P*2_1_2_1_2_1_
Unit cell parameters (Å)	*a* = 61.6, *b* = 80.0*, c* = 124.1	*a* = 62.1, *b* = 80.7*, c* = 124.7
Wavelength (Å)	0.98	0.99
Resolution Range (Å) ^a^	49.0–3.3 (3.5–3.3)	49.3–2.1 (2.2–2.1)
Unique reflections	9829	37 397
Multiplicity	38.7	6.4
Completeness (%) ^a^	99.8 (98.9)	100.0 (99.8)
I/σ(I) ^a^	21.2 (5.6)	13.8 (2.9)
*R*_sym_ (%)^a^	21.8 (85.5)	6.0 (57.3)
$ {\rm CC}\left({\frac{1}{2}} \right)^{\rm a}$	0.99 (0.95)	0.99 (0.65)

**Refinement**		
Resolution Range		49.3–2.1
*R*_free_ (%)		23.7
*R*_work_ (%)		20.6
r.m.s.d. bond (Å)		0.008
r.m.s.d. angle (º)		1.08
B-factor protein (Å^2^)		48.0

**Ramachandran values ^b^**		
Favored (%)		98.0
Allowed (%)		2.0
Outliers (%)		0

^a^Parantheses indicate values in outermost shell.

^b^Values were calculated using the program Molprobity (55).

We compared the heterodimerization surfaces of SMG5 and SMG7 with the same structural elements of SMG6. The 14–3–3-like domains of *C. elegans* SMG5 and SMG7 interact with a major hotspot at their α4 helices, which approach closely particularly due to glycine residues that are present at the equivalent position in the two proteins (Gly91 and Gly80 in *C. elegans* SMG5 and SMG7, respectively and Gly100 in human SMG7) (Figure 3D and Supplementary Figure S3) (42). Mutation of these glycine residues to glutamic acid has been shown to impair the SMG5–7 TPR interaction (42). In the structure of human SMG6, an aspartic acid (Asp683) is present at the equivalent position (Figure 3D and Supplementary Figure S3) and would be incompatible with the dimerization observed in SMG5–7 TPR. In addition, the interaction interface in the *C. elegans* SMG5–7 TPR structure is characterized by several hydrophobic contacts. One such contact involves a methionine residue in the α2 helix of SMG7 (Met30). The equivalent residue in SMG6 is a glutamate (Glu624) that would be incompatible with an analogous hydrophobic environment (Supplementary Figure S3). The structural analysis thus rationalizes why SMG6 TPR does not form homo- or heterodimers like the SMG5–7 TPRs, as small differences on the surface of the 14–3–3-like domain would result in steric and electrostatic clashes in a putative dimer.

### SMG6 binds UPF1 independently of phosphorylation

The 14–3–3-like domain of SMG6 has a similar arrangement of residues at the phosphoserine binding site as compared to SMG5 and SMG7. Many of the residues lining this site are conserved among the three proteins (Figure 3E). A detailed analysis indicates that the phosphoserine binding site in SMG6 is more similar to that of SMG5 than SMG7. A conserved lysine residue at this site in SMG7 (Lys 66 in the human protein) is replaced by a conserved tyrosine in both SMG6 (Tyr649, human numbering) and SMG5 (Tyr66, *C. elegans* numbering) (Figure 3E and Supplementary Figure S3). Thus, subtle differences in this pocket might modulate the binding of TPR proteins to phospho-UPF1. Since SMG6 does not heterodimerize with SMG7, we asked whether there might be an additional UPF1 binding site in another part of SMG6. To this end, we expressed and purified SMG6fl from HEK 293T cells and performed GST pull-down assays with *in vitro* phosphorylated (or mock-phosphorylated) GST-UPF1fl (Figure 1C). In contrast to SMG6 TPR in isolation, SMG6fl interacted with phosphorylated UPF1fl (Figure 1C, lane 7, compare with lane 5). Unexpectedly, SMG6fl interacted with UPF1fl also in the absence of phosphorylation (Figure 1C, lane 8).

**Figure 4.**
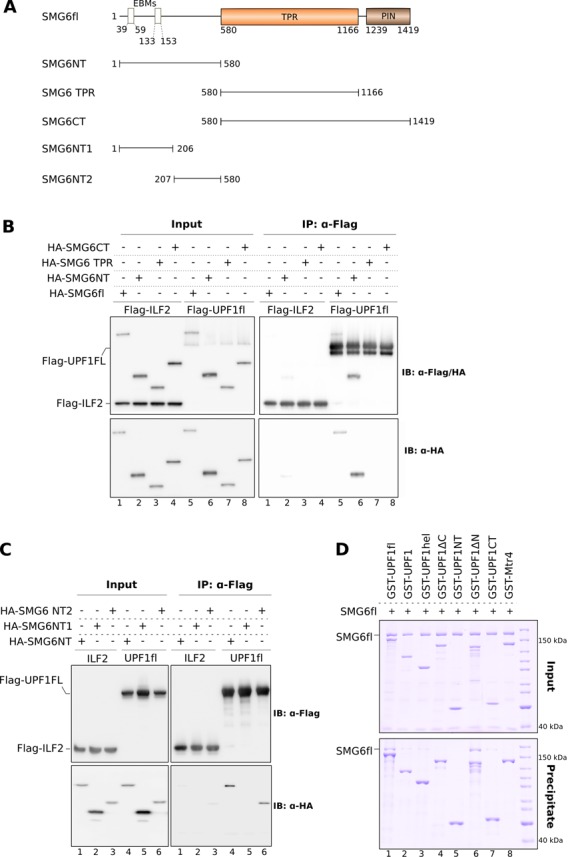
The N-terminal domain of SMG6 encompasses multiple discrete protein–protein interaction motifs. (**A**) Schematic representation of the SMG6 constructs designed to map the binding site of UPF1 on SMG6. (**B**) co-IP assays of Flag-UPF1fl and the indicated HA-SMG6 constructs (SMG6fl, SMG6NT, SMG6 TPR and SMG6CT). SMG6 constructs were co-transfected with UPF1 in HEK-293T cells. Cell lysates were subjected to IP using an anti-Flag antibody. 2% of the total cell lysate of every sample was used as the input. Inputs and precipitates were analyzed by SDS-PAGE and immuno blotting using the indicated antibodies and are depicted on the left and right panels, respectively. Flag-ILF2 was used as a negative control in this and other co-IP experiments, which were carried out in the absence of RNase A. Only the N-terminal domain of SMG6 was precipitated by Flag-UPF1 in a manner similar to SMG6fl, indicating the presence of a UPF1 interaction motif within this region. (**C**) Co-IP assays of Flag-UPF1fl and HA-tagged SMG6 N-terminal constructs (SMG6NT, SMG6NT1 and SMG6NT2). The experiment was performed as described above. The phospho-independent UPF1 interaction motif of SMG6 localized to a stretch that is proximal to the TPR domain and distinct from the EBMs located within the first 160 residues. (**D**) GST pull-down assays of different GST-UPF1 constructs (described in Figure 2A) and SMG6fl. The pull-downs and analysis were carried out as described in Figure 1C. The inputs and precipitates are shown on the top and bottom panels, respectively. Both the CH-helicase core and the C-terminal tail of UPF1 are important for SMG6 recognition.

In order to determine the domain of SMG6 that mediates additional interactions with UPF1, we generated truncation constructs of human SMG6 encompassing the N-terminal domain (SMG6NT, residues 1–580), the TPR domain (SMG6 TPR, residues 580–1166) or the TPR and PIN domains together (SMG6CT, residues 580–1419) (Figure 4A). These constructs contained an N-terminal hemagglutinin (HA) tag and were co-expressed in HEK 293T cells together with Flag-UPF1fl or Flag-ILF2 (used as a negative control). We carried out co-IP assays with an anti-Flag antibody (Figure 4B). With the caveat that only a small proportion of the over-expressed UPF1 might be phosphorylated in these conditions, neither SMG6 TPR nor SMG6CT interacted with UPF1fl (Figure 4B, right panels, lanes 7 and 8). In contrast, SMG6NT recapitulated the binding of SMG6fl to UPF1 (Figure 4B, right panels, lanes 5 and 6). We concluded that the N-terminal region of SMG6 contributes to its binding of UPF1.

### The N-terminal region of SMG6 interacts with the helicase domain and C-terminal tail of UPF1

We proceeded to narrow down the regions responsible for the phosphorylation-independent interaction of SMG6 and UPF1. The SMG6NT region is predicted to be largely unstructured (Supplementary Figure S4) and cannot be expressed as a soluble protein in *E. coli*. The only function known at present for this region of SMG6 resides in the EBM sequences that are present within the N-terminal 160 residues. We designed two truncation constructs spanning non-overlapping segments of SMG6NT: SMG6NT1 (residues 1–206), which contains both EBMs, and SMG6NT2 (207–579) covering the low-complexity stretch proximal to the TPR domain (Figure 4A). As before, we co-expressed these HA-tagged SMG6 constructs with Flag-UPF1fl in HEK 293T cells and performed co-IP assays with an anti-Flag antibody. We observed that SMG6NT2 bound UPF1 in a manner similar to SMG6NT (Figure 4C, lanes 4 and 6), while SMG6NT1 did not exhibit significant binding toward UPF1 (Figure 4C, lane 5). We concluded that the N-terminus of SMG6 interacts with UPF1 via an unstructured stretch that is proximal to the TPR domain and is distinct from the EJC-binding region (SMG6NT2).

We next performed GST pull-down assays with different GST-UPF1 constructs (Figure 2A) to identify the region of UPF1 that mediates the interaction with the SMG6 N-terminus. Since we could not purify SMG6NT2 in sufficient quantity and purity, we used SMG6fl as a prey for the GST pull-down assays (Figure 4D). We found that the binding of UPF1fl to SMG6 was recapitulated only by UPF1ΔN, which contains both the helicase region and the following C-terminal tail (Figure 4D, lane 6, compare with lane 1). In isolation, the UPF1 helicase region (UPF1hel) and the C-terminal tail (UPF1CT) independently showed a weak interaction with SMG6fl (Figure 4D, lanes 3 and 7). We concluded that the binding region for the SMG6 N-terminus maps to the helicase domain and C-terminal tail of UPF1. However, unlike UPF2, binding of SMG6 to UPF1 did not affect its catalytic (ATPase) activity (Supplementary Figure S4B).

### The UPF and EJC complexes can assemble with SMG6 and SMG5–SMG7 with concurrent interactions

**Figure 5.**
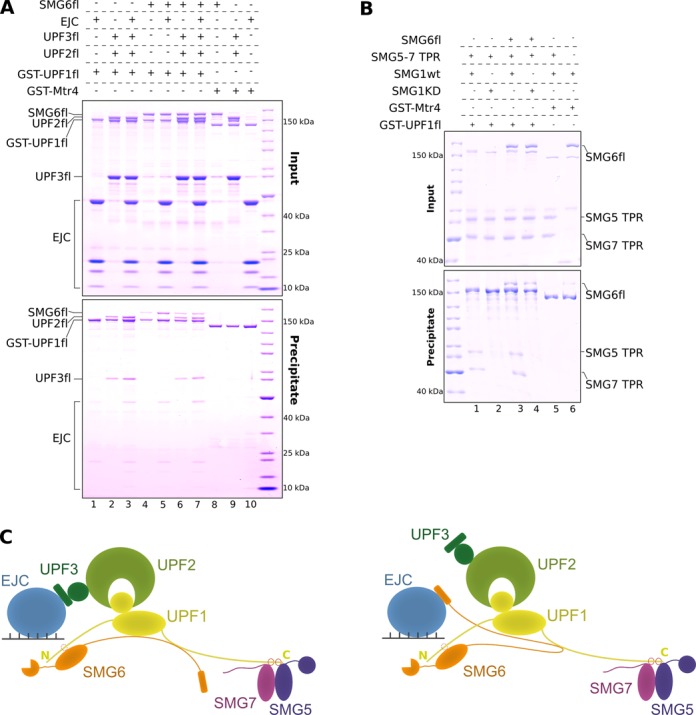
Assembly of complexes of multiple NMD factors in the presence of SMG6. (**A**) GST pull-down assays of GST-UPF1fl and SMG6fl in the presence of the NMD factors UPF2, UPF3 and the EJC. The pull-down was performed as described in Figure 1C. The inputs and precipitates were analyzed on 4–12% Bis-Tris gels (Invitrogen) and are depicted in the top and bottom panels, respectively. SMG6fl simultaneously interacted with UPF1 (even in the presence of UPF2 and UPF3) and the EJC, using its two distinct interaction motifs. The assembly of a large NMD complex consisting of SMG6, UPF1, UPF2, UPF3 and EJC is shown in lane 7 (see also Supplementary Figure S5). (**B**) GST pull-down assays of GST-UPF1fl (treated with active SMG1 wt or inactive SMG1 KD mutant) and SMG6fl and SMG5–7 TPR. The pull-downs and analysis were carried out as in Figure 1C. Top and bottom panels depict inputs and precipitates, respectively. Upon phosphorylation, GST-UPF1 simultaneously interacted with SMG6fl and SMG5–7 TPR, indicating that binding of the different TPR proteins to the C-terminus of UPF1 can occur concomitantly. (**C**) Model depicting the possible transient SMG6–UPF–EJC complexes assembled in the course of NMD, recapitulating the results from this study and previous reports. UPF1 (colored yellow) is depicted as a bilobal structure, with the small circle representing the N-terminal CH domain and the large circle representing the ATPase core. N-and C-terminal extensions of UPF1 are shown as yellow lines and phosphorylation sites therein are depicted as red open circles. UPF2 (in light green) binds the CH domain of UPF1 and UPF3 (colored dark green), forming the UPF complex. The TPR domains of SMG5 and SMG7 (colored purple and magenta) bind the C-terminal phospho sites of UPF1, while SMG6 TPR (colored orange) is thought to bind the N-terminal phospho site of UPF1. The N-terminus of SMG6 mediates a strong interaction with UPF1 and is crucial for its recruitment to the UPF–EJC complex. The interaction between UPF1 and the EJC (colored blue) can either be bridged by the EBM of UPF3 (left panel) or by the EBMs of SMG6 (right panel).

The contacts between UPF1 and SMG6 that we identified by *in vitro* pull-down assays and co-IP experiments raised predictions and questions in the context of the known NMD interaction network. It is well established that UPF1 uses the CH domain to bind UPF2 (62). UPF2 in turn binds the N-terminal RRM domain of UPF3 to form the ternary UPF complex (29,33,63). The observation that the CH domain of UPF1 is not required for SMG6 binding (Figure 4D, lane 4) indicates that UPF1 could bind UPF2–UPF3 and SMG6 at the same time. In pull-down assays, GST-UPF1fl could not only co-precipitate UPF2–UPF3 and SMG6 independently (Figure 5A, lanes 2 and 4), but also together to form a SMG6–UPF1–UPF2–UPF3 complex (Figure 5A, lane 6). Moreover, SMG6 uses different segments in its N-terminal region to bind UPF1 and the EJC, suggesting that these interactions could occur concomitantly. In pull-down assays, GST-UPF1fl indeed co-precipitated the EJC in the presence of SMG6fl to form an EJC–SMG6–UPF1 complex (Figure 5A, lane 5).

The UPF complex is known to bind the EJC via the C-terminal region of UPF3 (29,31–33), forming the seven-subunit EJC–UPF assembly (see also in the pull-down assay, Figure 5A, lane 3). The EBMs present in the C-terminal tail of UPF3 and the EBMs in the N-terminal region of SMG6 bind the EJC in a mutually exclusive manner, suggesting that the recruitment of SMG6 and the UPF complex to the EJC are separate steps in the NMD pathway (45). However, from the EJC–SMG6–UPF1 and SMG6–UPF1–UPF2–UPF3 interactions discussed above (Figure 5A, lanes 5 and 6), it is possible to envisage the formation of an EJC→SMG6→UPF1→UPF2→UPF3 complex or of an EJC→UPF3→UPF2→UPF1→SMG6 complex (where → represents a direct protein–protein interaction). In these large complexes, either SMG6 or UPF3 would bridge the interaction between the EJC and UPF complexes (Figure 5C, right and left panels, respectively). Indeed, GST-UPF1fl co-precipitated SMG6, UPF2, UPF3 and the EJC proteins to form an EJC–SMG6–UPF1–UPF2–UPF3 complex (Figure 5A, lane 7). Assembly of the large complex with the RRM domain of UPF3, which is capable of binding UPF2–UPF1 but not recruiting the EJC, confirms the role of SMG6 as a bridge between UPF1 and the EJC (Supplementary Figure S5, lanes 3 and 7).

Finally, the finding that SMG6 binding to unphosphorylated UPF1 requires both the helicase domain and C-terminal tail raises the question of whether this interaction is mutually exclusive with the binding of SMG5–7 TPR to the phosphorylated C-terminal tail of UPF1. In the presence of SMG1 wt, GST-UPF1fl co-precipitated both SMG6fl and SMG5–7 TPR (Figure 5B, lane 3), suggesting that UPF1 can, in principle, recruit SMG6 and SMG5–7 concomitantly.

## CONCLUSIONS

Much like canonical 14–3–3 dimers, the dimeric nature of SMG5–SMG7 is important for their function (35,42,44). The *in vitro* data with recombinant proteins reported here recapitulate the results from cell-based assays (38,42), converging on the view that the 14–3–3-like domain of SMG7 contains a strong phosphoserine binding site that binds the segment centered at phosphorylated Ser1096. In analogy with 14–3–3 proteins (64), the motif that binds SMG7 is the ‘gatekeeper’ whose presence is required for binding. The 14–3–3-like domain of SMG5, instead, contains a weaker phosphoserine binding site that binds a segment centered at Ser1116. The linker connecting Ser1096 and Ser1116 is about the twice the length as compared to the minimal distance found in phosphorylated targets of canonical 14–3–3 proteins (65). This observation fits with the finding that the 14–3–3-like domains of SMG5 and SMG7 dimerize in a back-to-back orientation that positions the two phosphoserine binding sites at roughly twice the distance as compared to canonical side-by-side 14–3–3 dimers (42).

In contrast to canonical 14–3–3 proteins, the SMG6 TPR is a monomer in solution. At the structural level, subtle changes at the putative dimerization interface rationalize the inability of SMG6 TPR to dimerize with either SMG5 or SMG7 TPRs. Subtle differences also exist at the phosphoserine binding site of SMG6, making it more similar to the weaker site of SMG5 rather than to the stronger site in SMG7. The dominant binding site for the interaction with UPF1 resides in a region upstream of the 14–3–3-like domain of SMG6 and is thus provided within the same protein. In the case of SMG6 binding, the ‘gatekeeper’ is a composite site that binds the helicase core and the C-terminal tail. Notably, this interaction between UPF1 and SMG6 is phosphorylation independent. In light of reports that phosphorylated Thr28 in UPF1 is important for binding SMG6 in cells (38), it is possible that this site provides a secondary, low affinity interaction to the 14–3–3-like domain of SMG6 that could act in synergy with the dominant site.

The interaction data we obtained indicate that phosphorylated UPF1 binds SMG5–SMG7 and SMG6 directly and simultaneously, and is part of a larger complex that include UPF2, UPF3 and the EJC (Figure 5C). These concomitant interactions are stable enough to withstand *in vitro* reconstitutions, but are likely to occur in a transient fashion and in a sequential manner during NMD. The results raise a new mechanistic hypothesis on the spatial and temporal dimensions of the NMD interaction network. For example, the recruitment of SMG6 near the EJC might not necessarily occur after UPF3 dissociation (45). It is possible that SMG6 binding to UPF1 increases the effective local concentration of its two EBMs, allowing them to dissociate the UPF3 from the EJC. Furthermore, SMG1 can phosphorylate UPF1 efficiently, although studies with *C. elegans* mutants have shown that phosphorylation of UPF1 by SMG1 also requires UPF2 and UPF3 (34). The requirement of UPF2 and UPF3 in the *in vivo* studies might simply reflect the step in the pathway when phosphorylation takes place. Consistent with these notions, it has been shown that SMG1 associates with post-spliced mRNAs via UPF2 and via a subunit of the EJC and that UPF2 dissociates SMG1 from its negative regulators SMG8 and SMG9 (34,40). Thus, UPF1 might be phosphorylated in the context of the EJC–UPF assembly *in vivo* because the efficient recruitment of active SMG1 occurs in the context of this assembly.

The phosphorylation and dephosphorylation cycle of UPF1 appears to be a relatively recent development in evolution. In yeast, UPF1 lacks the unstructured SQ-containing tails. However, a recent mass-spectrometry study identified 11 novel phosphorylation sites in yeast Upf1p (66). Furthermore, a putative ortholog of SMG7, Ebs1p, was shown to function in the yeast NMD pathway possibly by recruiting Upf1p to P-bodies (67,68). Our finding that the helicase domain of human UPF1 binds SMG6 in a phosphorylation-independent manner raises yet another interesting possibility that yeast Upf1p might use a similar interaction mechanism to help recruiting another factor and prompting RNA degradation. The expectation is that the mechanisms of NMD in different species will eventually be merged in a unified model, not only for the upstream steps involving PTC-mRNA recognition but also for the downstream steps involving the targeting to degradation.

## ACCESSION NUMBERS

The coordinates and the structure factors of SMG6 TPR have been deposited in the Protein Data Bank with the accession code 4UM2.

## SUPPLEMENTARY DATA

Supplementary Data are available at NAR Online.

SUPPLEMENTARY DATA
