# Supplementary material for: Phospho-dependent and phospho-independent interactions of the helicase UPF1 with the NMD factors SMG5–SMG7 and SMG6
Source: Nucleic Acids Res. 2014 Jul 10;42(14):9447–60. doi: 10.1093/nar/gku578 (PMC4132714; doi:10.1093/nar/gku578)
Supplement: SUPPLEMENTARY DATA [file supp_42_14_9447__index.html]

Phospho-dependent and phospho-independent interactions of the helicase UPF1 with the NMD factors SMG5–SMG7 and SMG6 — Phospho-dependent and phospho-independent interactions of the helicase UPF1 with the NMD factors SMG5–SMG7 and SMG6 — SUPPLEMENTARY DATA 

# Phospho-dependent and phospho-independent interactions of the helicase UPF1 with the NMD factors SMG5–SMG7 and SMG6

## SUPPLEMENTARY DATA

**Files in this Data Supplement:**

- Supplementary Data
