# Supplementary material for: Phospho-dependent and phospho-independent interactions of the helicase UPF1 with the NMD factors SMG5–SMG7 and SMG6
Source: Nucleic Acids Res. 2014 Jul 10;42(14):9447–60. doi: 10.1093/nar/gku578 (PMC4132714; doi:10.1093/nar/gku578)
Supplement: SUPPLEMENTARY DATA [file supp_gku578_nar-00929-a-2014-File008.pdf]

Supplementary Information

Phospho-dependent and phospho-independent recognition of the helicase UPF1 by the NMD factors SMG5-SMG7 and SMG6

Sutapa Chakrabarti, Fabien Bonneau, Steffen Schüssler, Elfriede Eppinger and Elena Conti*

Max Planck Institute of Biochemistry
Structural Cell Biology Department
Am Klopferspitz 18
D-82152 Martinsried
Germany

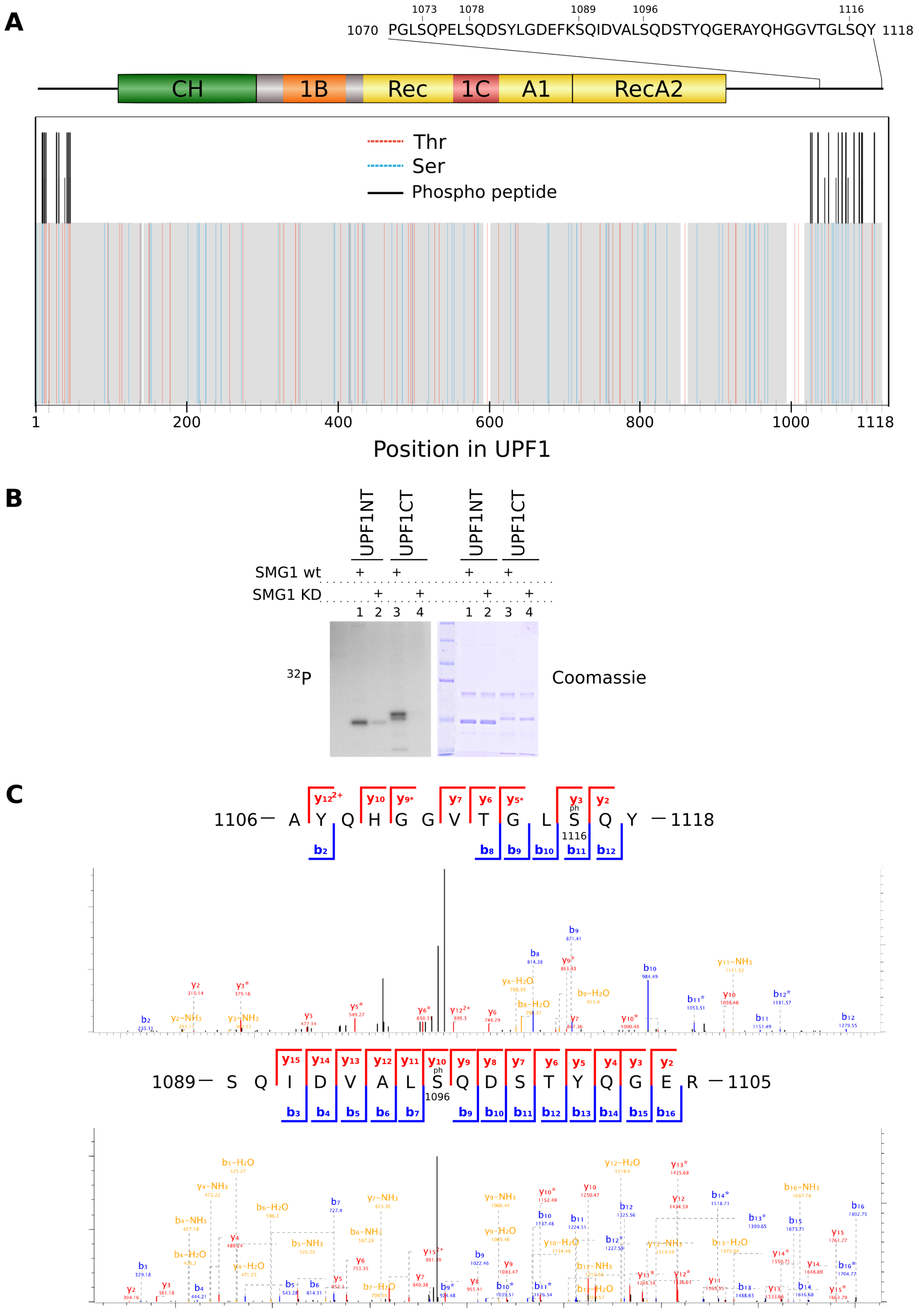

Figure S1.

(**A**) Phospho-peptide and LC-MS/MS mapping of the SMG1 phosphorylation sites on UPF1. The gray regions indicate the total peptide coverage within UPF1, upon

treatment with a combination of different proteases (trypsin, Asp-N, chymotrypsin and thermolysin). Orange and blue bars indicate the position of every threonine and serine residue, respectively. The black bars denote the identified phospho-sites, with half-length bars corresponding to sites that had identification and localization scores lower than the default 75% threshold. Mock phosphorylated UPF1 gave identical peptide coverage and no phospho-peptides were identified. The sequence of residues 1070-1118 of UPF1 and the positions of the SQ motifs therein are indicated.

(**B**) In vitro kinase assays (using γ-^{32}P ATP) of the UPF1 N- and C-terminal constructs (UPF1NT and UPF1CT) with purified SMG1 proteins (left panel). The corresponding Coomassie-stained gel of the radioactive kinase assay is shown in the right panel. The isolated ends of UPF1 are efficiently phosphorylated by SMG1 wt but not by SMG1 KD. The experiment was performed as in Figure 1C (described in the main text).

(**C**) MS/MS spectra for phosphorylated GST-UPF1CT4. The top panel denotes fragmentation of the peptide harboring the S1116 site and confirms the presence of a phospho-group on S1116. The bottom panel corresponds to the peptide encompassing the S1089 and S1096 sites and confirms the presence of a phospho-group on S1096.

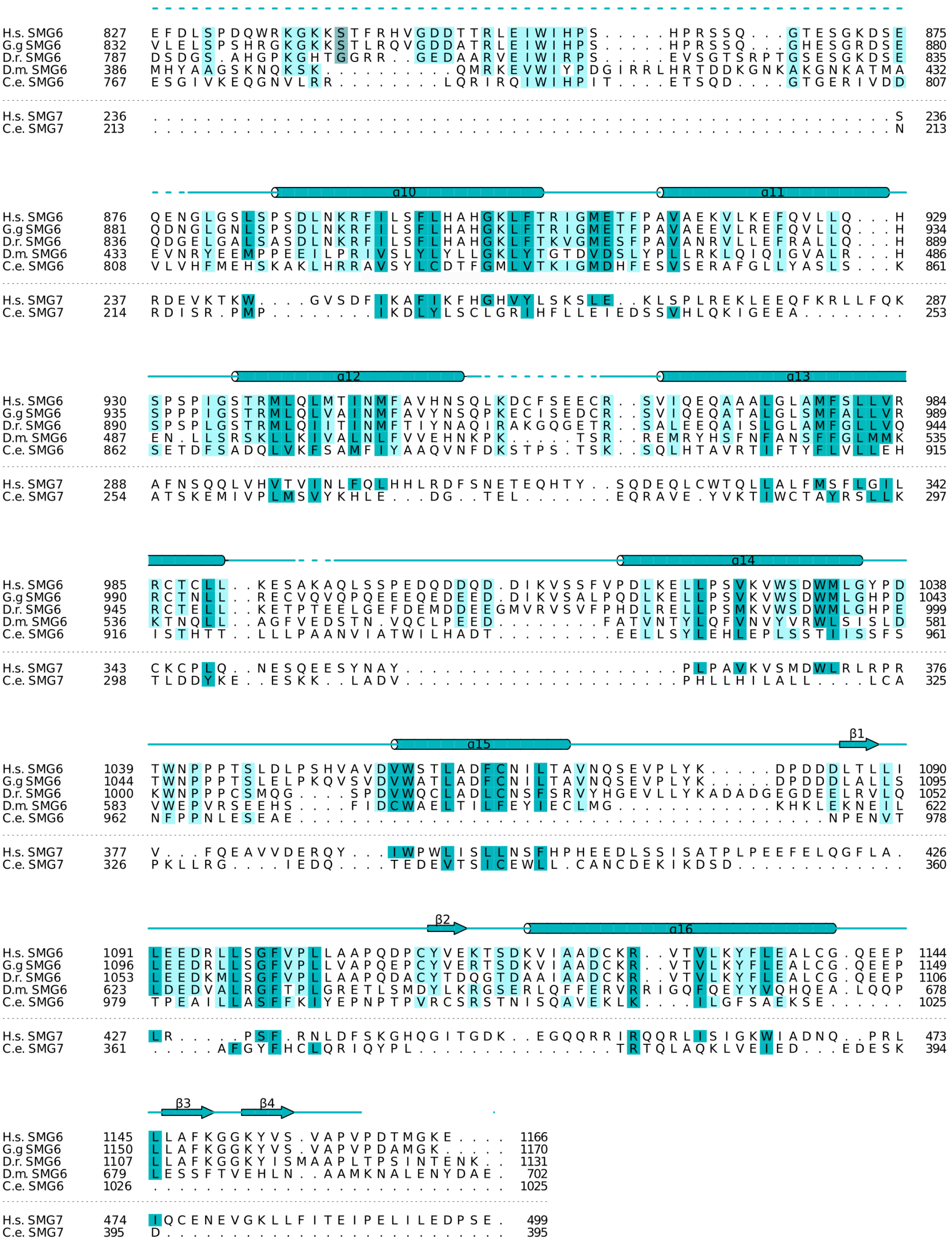

Figure S2

Structure-based sequence alignment of the helical hairpins domains of SMG6 from *H. sapiens* (H.s.), *G. gallus* (G.g.), *D. rerio* (D.r.), *D. melanogaster* (D.m.), C. *elegans* (C.e.) and the helical hairpins domains of SMG7 from *H. sapiens* (H.s.)

and C. *elegans* (C.e.). The secondary structural elements of H.s. SMG6 are represented as cylinders (α-helices) or arrows (β-strands) and colored teal, as per Figure 3C. Dashed lines represent disordered regions in the structure. H.s. SMG6 has a long loop preceding the helical hairpins domain, which is disordered in our structure. Conservation was determined based on the chemical property of amino acids. Residues conserved between SMG6 and SMG7 are shaded in dark teal while residues conserved only across SMG6 orthologues are shaded in cyan. The helical hairpins domain of SMG5 diverges considerably in its sequence and architecture from those of SMG6 and SMG7 and was omitted from the alignment.

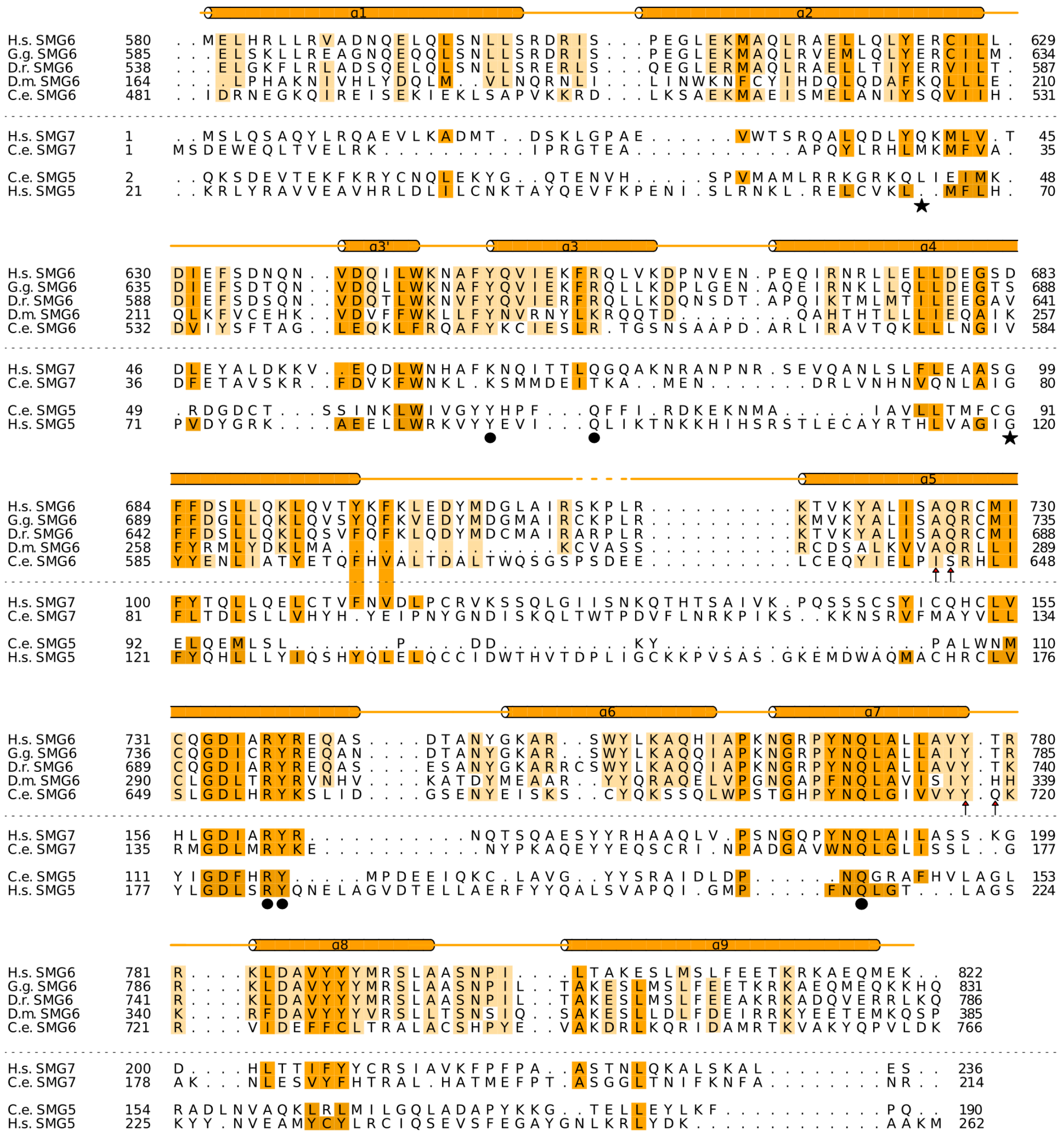

Figure S3

Structure-based sequence alignment of the TPR domains of SMG6 from *H. sapiens* (H.s.), *G. gallus* (G.g.), *D. rerio* (D.r.), *D. melanogaster* (D.m.), *C. elegans* (C.e.) and the TPR domains of SMG7 and SMG5 from *H. sapiens* (H.s.) and C. *elegans* (C.e.). The secondary structural elements of H.s. SMG6 are represented as cylinders (α-helices) or arrows (β-strands) and colored orange, in keeping with Figure 3C. Dashed lines represent disordered regions in the structure. Black circles indicate amino acids

in the TPR domain that are thought to bind the phospho-serine moiety (based on the structure of H.s. SMG7 TPR (1)), and black stars denote residues of SMG5 and SMG7 (only those mentioned in the text) that mediate interaction between the TPR domains of these two proteins (based on the structure of C.e. SMG5:7 TPR, (2)). Conservation was determined based on the chemical property of amino acids. Residues conserved among SMG6, SMG7 and SMG5 are shaded in dark orange while residues conserved only across SMG6 orthologues are shaded in light orange.

A

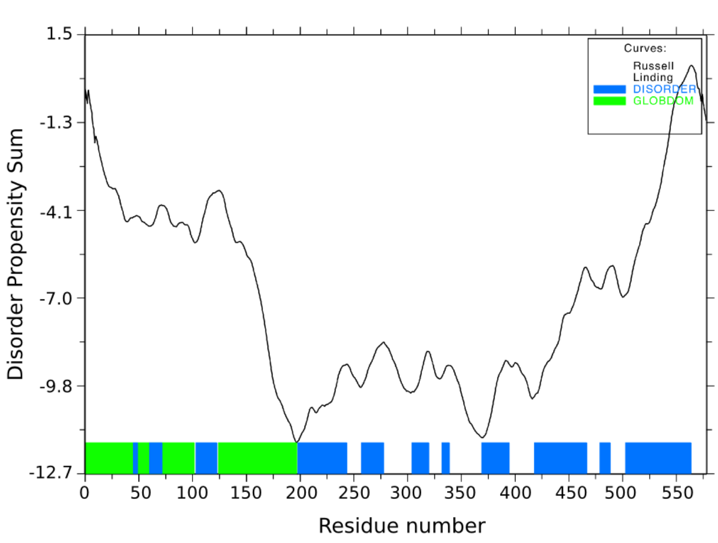

B

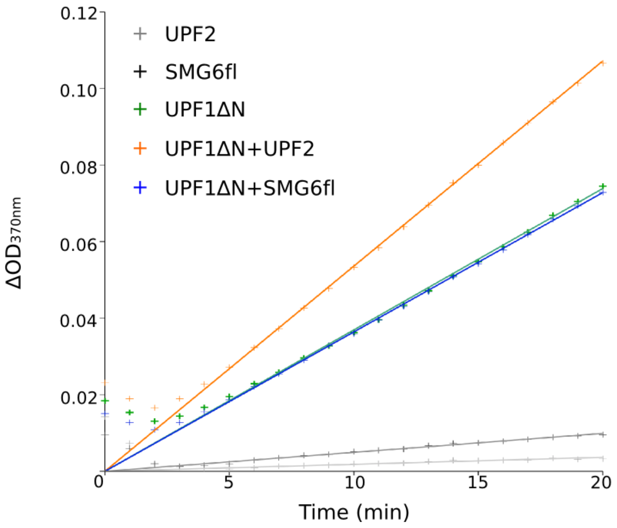

Figure S4

(**A**) Graphical representation of the intrinsic disorder propensity of residues 1-580 of human SMG6. Green bars indicate ordered/globular domains while blue bars denote disordered stretches. The disorder propensity was calculated using the GlobPlot tool, available at http://globplot.embl.de (3).

(**B**) RNA-dependent ATPase assay of UPF1ΔN, in the absence and presence of UPF2(761-1227) and SMG6fl, performed using a coupled phosphate-detection assay (4). Reaction trends were determined using the 5-15 minute time points. The catalytic activity of UPF1 is enhanced upon addition of UPF2 but not upon addition of SMG6.

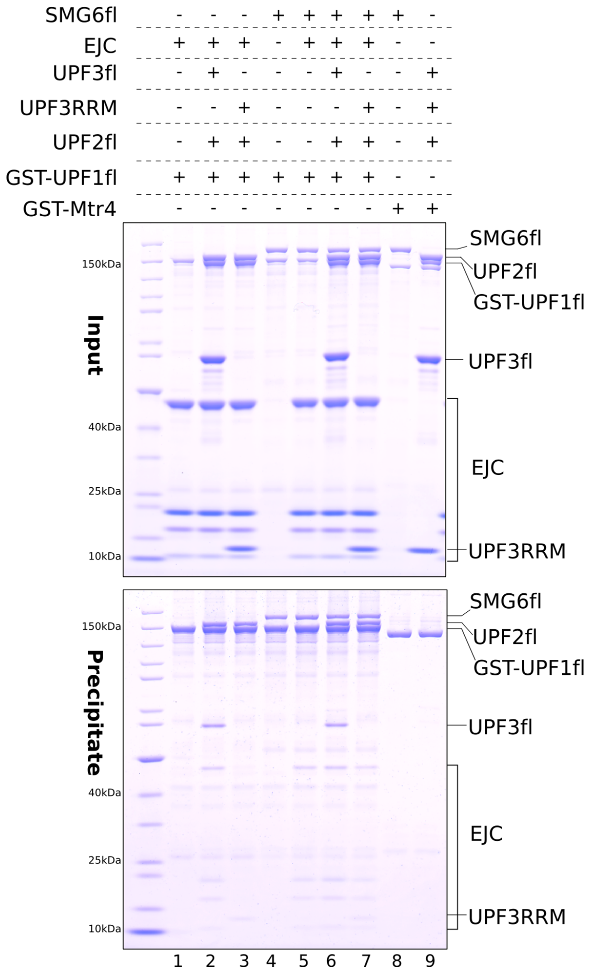

Figure S5

GST pull-down assays of GST-UPF1fl and SMG6fl in the presence of the EJC, UPF2 and two constructs of UPF3 (UPF3fl and UPF3RRM). The pull-down was performed as described in Figure 1C in the main text. The inputs and precipitates were analyzed on 4-12% Bis-Tris gels (Invitrogen) and are depicted in the top and bottom panels, respectively. Unlike UPF3fl, UPF3RRM is incapable of recruiting the EJC to a UPF1-UPF2 complex (lane 3, compare with lane 2). However, a complex containing UPF1-UPF2-UPF3RRM and the EJC is assembled in the presence of SMG6 (lane 7), indicating the role of SMG6 in bridging the EJC and UPF1 using its two distinct interaction motifs (EBMs and UPF1-interacting motif).

Supplementary Methods

Phospho-peptide mapping

1µg UPF1fl was incubated for 3 hours at 30˚C with or without SMG1 for in presence of 1 mM ATP and 2% phosphatase inhibitor cocktail 2 and 3 (Sigma), in a buffer containing 25 mM MOPS pH 7.2, 5 mM $MgCl_2$, 10 mM β-Glycerophosphate and 50 µg/mL BSA. Proteins were separated on a 7.5% SDS-polyacrylamide gel and stained with Coomassie. Bands corresponding to UPF1 were cut, in-gel digested with either AspN, Chymotrypsin, Chymotrypsin/LysC, Thermolysin, or Trypsin/LysC. Peptides mixtures were analysed on a LTQ Orbitrap XL (Thermo Scientific). Peptide identification was performed with MaxQuant version 1.3.0.5 (5). MaxQuant enables high peptide identification rates, individualized p.p.b.-range mass accuracies and proteome-wide protein quantification, allowing for 10 missed cleavages and a minimal peptide length of 5 residues. The search space was composed of non-redundant databases for human and *E. coli* proteins. In the human protein database, sequences for endogenous human UPF1 were removed and replaced by the His-tagged version of the full-length UPF1 used in our assay.

ATPase assay

40 nmoles of MESG (2-amino-6-mercapto-7-methylpurine ribonucleoside) and 0.05U of purine-nucleoside phosphorylase (components of the EnzChek® Phosphate Assay kit, Life technologies) were pre-incubated with 2 µg poly-U RNA in 1X ATPase buffer (50 mM MES pH 6.5, 50 mM potassium acetate, 5 mM magnesium di-acetate, 2 mM DTT) at 30°C for 20 minutes, following which 10 pmoles of UPF1ΔN and 20 pmoles of UPF2(761-1227) or SMG6fl were added to the indicated samples. The

reaction was initiated by the addition of 10 mM ATP. Generation of 2-amino-6-mercapto-7-methylpurine from MESG and phosphate (released from ATP hydrolysis) was detected by measuring absorbance at 370 nm on a Genios Pro (Tecan). The reaction was allowed to proceed for 20 minutes and A_{370} was measured at 60-second intervals.

Supplementary References

1. Fukuhara, N., Ebert, J., Unterholzner, L., Lindner, D., Izaurralde, E. and Conti, E. (2005) SMG7 is a 14-3-3-like adaptor in the nonsense-mediated mRNA decay pathway. *Mol Cell*, **17**, 537-547.
2. Jonas, S., Weichenrieder, O. and Izaurralde, E. (2013) An unusual arrangement of two 14-3-3-like domains in the SMG5-SMG7 heterodimer is required for efficient nonsense-mediated mRNA decay. *Genes Dev*, **27**, 211-225.
3. Linding, R., Russell, R.B., Neduva, V. and Gibson, T.J. (2003) GlobPlot: Exploring protein sequences for globularity and disorder. *Nucleic Acids Res*, **31**, 3701-3708.
4. Webb, M.R. (1992) A continuous spectrophotometric assay for inorganic phosphate and for measuring phosphate release kinetics in biological systems. *Proc Natl Acad Sci U S A*, **89**, 4884-4887.
5. Cox, J. and Mann, M. (2008) MaxQuant enables high peptide identification rates, individualized p.p.b.-range mass accuracies and proteome-wide protein quantification. *Nat Biotechnol*, **26**, 1367-1372.
